# Assessment of pain symptoms and quality of life using the International Spinal Cord Injury Data Sets in persons with chronic spinal cord injury

**DOI:** 10.1038/s41394-019-0178-8

**Published:** 2019-04-15

**Authors:** Katie Gibbs, Andrew Beaufort, Adam Stein, Tung Ming Leung, Cristina Sison, Ona Bloom

**Affiliations:** 1 The Feinstein Institute for Medical Research, New York, USA; 2 Department of Physical Medicine and Rehabilitation, Northwell Health, New York, USA; 3 Biostatistics Unit, Northwell Health, New York, USA; 4 Department of Molecular Medicine, Zucker School of Medicine at Hofstra-Northwell, New York, USA

**Keywords:** Chronic pain, Translational research

## Abstract

**Introduction:**

Traumatic spinal cord injury (SCI) triggers complex changes that can negatively impact health and quality of life. The International SCI Data Sets were developed to enable more comparable data collection on the complex sequelae of SCI across studies. This should facilitate progress in mechanistic understanding and improving treatments of SCI.

**Study design:**

Prospective observational pilot study.

**Objectives:**

To collect data on pain symptoms and quality of life (QoL) in adults living with chronic SCI.

**Setting:**

Academic medical center, New York, USA.

**Methods:**

The International SCI Basic Pain and Qol Data Sets were used to collect data from participants with chronic SCI (*N* = 31) at 2 study visits held 6 months apart. The QoL Data Set was also used to collect data from able-bodied persons of similar age and gender distribution (*N* = 28).

**Results:**

Most participants with SCI had multiple types and locations of pain problems at both study visits, despite reported being treated for pain. At both visits, the worst pain problem type was nociceptive, followed by neuropathic, which was typically rated of higher intensity. QoL scores were significantly lower across all domains of the data set in persons with SCI than able-bodied persons. Persons with pain tended to have lower QoL scores, although this trend was not significant.

**Conclusions:**

This study demonstrates the presence, complexity and stability of pain symptoms refractory to treatment and lower quality of life ratings in persons with chronic SCI.

**Sponsorship:**

Grants from the Craig H. Neilsen Foundation, New York Empire Clinical Research Program, New York State Spinal Cord Injury Research Board.

## Introduction

Pain is recognized as a most common medical consequence of traumatic spinal cord injury (SCI), with prevalence rates estimated to be ~60% [1, 2]. For many individuals with SCI, pain severity remains consistent over time. In data from the NIH SCI Model Systems database, on a scale of 0–10, where 0 is none and 10 is the worst pain, the mean score for pain severity after injury was 4.2 at year 1, 4.5 at year 10, 4.3 at year 20, and 4.2 at year 40 [3]. Pain interferes with quality of life (QoL); in the Model Systems database, at 1 year after injury, interference of pain with work was: “not at all” for 18%, “a little bit” for 21%, “moderately” for 14%, “quite a bit” for 12.8%, and “extremely” for 6.4% of respondents [3]. Data from the Model Systems also show that pain interference increased with time: no pain interference was lowest at 1 year and highest at 25 years after SCI [3].

With high prevalence, complexity, and persistence of pain in persons with SCI, Bryce, Ragnarsson and colleagues have had a longstanding effort to develop a reliable pain taxonomy to facilitate better mechanistic understanding and treatment [4–6]. Recently, common data elements were created to facilitate characterization of pain and other complex medical and psychosocial sequalae of SCI [7, 8]. The International SCI Pain Basic Data Set (ISCIPBDS) standardizes the characterization and reporting of SCI pain across studies [9]. It builds on the International SCI Pain Classification (ISCIP) to classify pain first by type (nociceptive, neuropathic, other, or unknown), subtype (nociceptive: musculoskeletal, visceral, or other, neuropathic: at or below level, or other unrelated to the injury) and then by pain source and/or pathology [10]. In accordance with ISCIP, ISCIPBDS includes questions about a person’s 3 worst pain problems, including: pain types and subtypes, locations, intensities, frequency, and duration, as well as some basic information on how pain interferes with activities of daily living, mood, sleep and social participation [9]. The ISCIPBDS was recently found to have good validity as a self-reported measure of pain [11]. A second version of the ISCIPBDS was simplified to facilitate use, eliminating some questions about pain chronicity, some pain interference questions and adding a category of “other” to pain type [12]. The ISCIPBDS also contains a few questions on pain interference to relate how pain impacts QoL. Satisfaction with QoL is also addressed in an independent International SCI QoL Basic Data Set, which has been shown to have good validity in the SCI population [13, 14].

Here, the main objective was to characterize symptoms of pain using the ISCIPBDS in adults with chronic SCI (≥1 year from initial injury). To characterize pain persistence and variability in the chronic phase of SCI, data were obtained at 2 study visits held 6 months apart. A secondary goal was to collect data using the International SCI QoL Basic Data set to examine potential inverse correlations between pain symptoms and satisfaction with QoL.

## Methods

This prospective, observational study was performed in an academic medical center in accordance with ethical standards and with approval from the local IRB. Inclusion criteria were: ≥18 years old, history of SCI at any level, traumatic SCI at least 1 year prior, American Spinal Injury Association Impairment Scale (AIS) grades A–D, as determined by a physiatrist board certified in SCI medicine. Exclusion criteria were: concurrent infection such as frank urinary tract infection as indicated by lab evidence (urinalysis, positive culture) and some clinical occurrence such as hematuria, fever; incontinence between catheterizations; pressure ulcers, cancer, chemotherapy, neutropenia, or autoimmune disease. Many, but not all, study participants received medical care at the study institution, so some relevant information, including medication lists, were more complete for some participants than for others. For example, although opioids are not currently recommended for pain treatment in persons with SCI and are not commonly prescribed at the study institution, some participants reported prescriptions for opioids when asked if they were taking medications for pain. Functional genomic and biochemical data from some of the same participants were published previously [15, 16]. A group of similar age able-bodied individuals (*N* = 28) were recruited for comparison.

To address persistence and variability of pain symptoms in chronic SCI, participants with SCI were asked to complete two study visits held six months apart. Most participants (81%) completed both visits. Pain data were collected using ISCIPBDS (v1.1 & 2.0). Since the numerical rating scale of pain intensity changed between versions, data were normalized as a percentage of the scale (1–10) used in version 2. Additional data (e.g., medications potentially related to pain management) was requested by interview from participants or abstracted from participants’ medical charts when available. QoL data were collected using the International SCI Quality of Life Basic Data set. Descriptive statistics (frequency distribution for categorical variables and mean, median, SD, minimum, and maximum for continuous variables) were calculated. Chi square and Fisher’s Exact tests were performed to test associations between pain, injury status, and mechanism of injury. Mann–Whitney Rank Sum test was performed to test if there was any difference in years after injury in persons with or without pain, and difference in average pain intensity and in QoL scales among participants with different SCI levels, neurological injury motor status, or mechanisms of injury. As this was an exploratory study, no corrections were made on multiple tests. However, the Dwass, Steel, Critchlow-Fligner (DSCF) correction for multiple levels comparison analysis was applied to the Rank Sum test if the overall analysis was significant. Box plots were used to visualize average differences between groups in Mann–Whitney Rank Sum test. Spearman correlations were calculated to examine associations of years after injury and QoL scales, and of average pain intensity and QoL scales. Potential differences in pain symptoms between study visits and potential correlations between pain symptoms and QoL were examined among SCI participants, where data were available. For comparison, QoL data were also collected from a group of able-bodied persons (*N* = 28). Statistical analyses were performed using SAS v9.3 (SAS Inc., Cary, NC) and Prism GraphPad 6 Software.

## Results

### Participant characteristics

Basic clinical and demographic information for participants is shown in Table 1. Participants (81% males) were individuals living with SCI for ≥1 year and had an average time from injury of 15.7 ± 2.3 years (mean ± SEM). The average age of participants with SCI was 55.0 ± 3 (mean ± SEM), and range was 21–80 years. The mechanisms of injury were: Fall (32%), Sports (32%), MVA (23%), and Other (13%). Most participants had cervical level injuries (58%), followed by thoracic (36%) and lumbar (6%).

**Table 1:** Participant demographics

	Mean ± SEM, Range
Age: years	
Able-bodied (AB) persons (*N* = 28)	48 ± 2, 23–66
Persons with chronic SCI (*N* = 31)	55 ± 3, 21–80
Gender: male	N
AB	22
SCI	22
Years post injury	15.7±2, 1–44
Mechanism of injury	N
Fall	10
MVA	7
Sports	10
Other	4
AIS grade	N
A	16
B	2
C	4
D	9
Level of injury	N
Cervical	18
Thoracic	11
Lumbar	2

### International SCI pain data set

The ISCIPBDS asks if respondents are receiving any treatments for any type of pain (neuropathic, nociceptive, or other) problems. Therefore, participants were asked by a study investigator if they were using any kind of pain management strategy. (Some study participants received medical care from the study institution and therefore more complete medication records were available for some participants.) A majority of participants (76% and 68% at visits 1 and 2, respectively) reported using multiple concurrent medications or therapeutic strategies that may have been related to pain management (Table 2). Pharmacological treatments that participants reported may have been related to pain management most commonly included pregabalin or gabapentin, followed by NSAIDS with or without aspirin, and others (Table 2). Other strategies were also reported (Table 2).

**Table 2:** Treatments related to pain management. Data were collected by interview with participants and supplemented, when available, by examination of their medical charts

	Participants (%)
A. Pharmacological	
Anti-epileptics (pregabalin, gabapentin)	32
NSAIDS with Aspirin	32
NSAIDS without Aspirin	19
Opioids (morphine, oxycodone, buprenorphine)	26
Benzodiazepines	10
Anti-depressants	6.5
Muscle relaxers	3
Epidural steroid injection	3
B. Non-pharmacological	
Physical therapy	13
Massage	3
Heat	3
Magnet therapy	3

Total percent exceeds 100%, due to participants using more than one therapeutic strategy

**Fig. 1:**
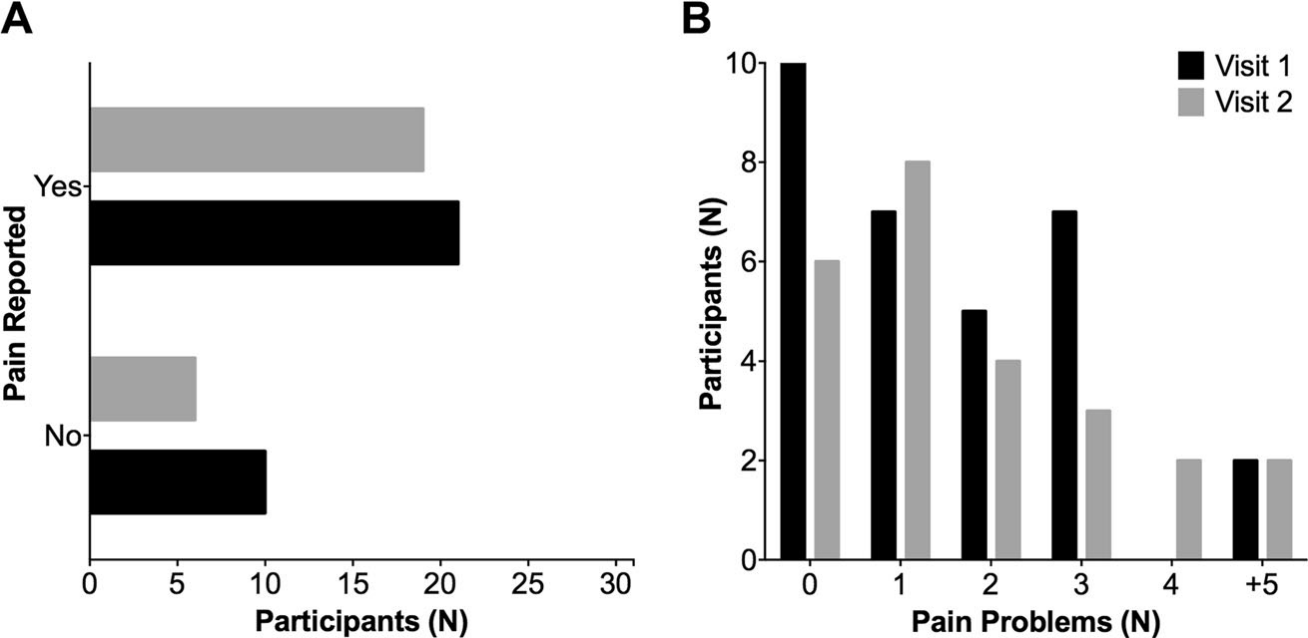
Pain Status and Number of Pain Problems: **a** *x*-axis shows the number of participants who reported pain (yes/no, *y*-axis). **b** *x*-axis shows the number of pain problems that were reported (0–4 or at least 5) by the number of participants indicated on the *y*-axis. Data are shown for visit 1 (black), visit 2 (gray)

At each study visit, a member of the study team asked participants about their pain symptoms as specified by the ISCIPBDS. The first question asked is if the participant has had any pain during the last seven days including today. Among participants, 67% and 76% had pain at visit 1 and 2, respectively (Fig. 1a). Most participants had more than one pain problem at each study visit (Fig. 1b). Among participants with pain, 47% had neuropathic and 53% had nociceptive types of pain problems. The next question asked is if the participant can rank the type of their three worst pain problems. At both study visits, the worst type of pain problem reported was nociceptive (53%) (Table 3a). At both visits 1 and 2, within the worst pain problem, the most common subtype of nociceptive pain was musculoskeletal (Table 3a). Neuropathic pain was characterized most commonly as below level (Table 3a). Data on location, type, intensity, and frequency of pain was then collected for the second and worst pain problems (Table 3a).

**Table 3:** Ranking and location of three worst reported pain problems. Data were collected by interview with participants on the three worst pain problems they experienced within the last seven days, by pain type and location

Pain problem by rank		1st		2nd		3rd	
	Visit	1	2	1	2	1	2
A. Pain types							
Pain type							
Nociceptive	Musculoskeletal	10	9	8	4	4	4
	Visceral	0	1	0	0	0	2
	Subtotal	10	10	8	4	4	6
Neuropathic	Below	5	5	1	3	2	0
	At	3	3	0	1	0	1
	Other	0	0	1	0	0	0
	Subtotal	8	8	2	4	2	1
Unknown		1	0	3	2	1	0
All types	Total	19	18	13	10	7	7
B. Pain locations							
Pain location							
Head		0	0	0	0	1	1
Neck/shoulders		6	5	3	2	2	3
Arm/hands		1	1	3	2	1	0
Frontal torso/genitals		4	3	2	1	1	1
Back		5	3	3	3	3	1
Buttocks/hips		1	1	0	1	0	0
Upper legs/thighs		3	4	2	1	0	1
Lower legs/feet		1	2	1	1	1	0
All locations	Total	21	19	14	11	9	7

**Fig. 2:**
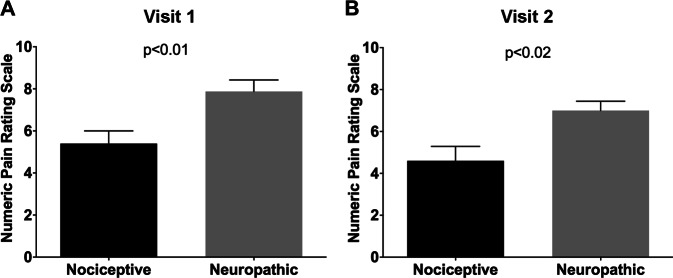
Pain intensity for the worst reported pain problem: the intensity of the worst reported pain problem (either nociceptive or neuropathic) by type for Visit 1 (**a**) and Visit 2 (**b**)

The pain intensity by pain type was reported according to a numerical rating scale of 0–10, where 0 is “no pain” and 10 is “pain as bad as you can imagine”. At visit 1, for the worst pain problem, the average pain intensity scores ranged from 1–10. Although more participants reported nociceptive as their worst type of pain problem, the average pain intensity scores were higher for the fewer participants, who reported neuropathic as their worst type of pain problem (Fig. 2). In version 1 of the ISCIPBDS, it then asked about duration and frequency of pain. This data were not collected consistently from participants here and is therefore not reported; this data collection was removed from ISCIPBDS version 2.

The ISCIPBDS asks respondents to identify their pain locations/sites for each of their three worst pain problems. The pre-specified location choices are shown in Table 3. Neck/shoulders was the most common location for the worst pain problem at visit 1 or 2 (Table 3b). This was followed by back, frontal torso/genitals and then upper legs/thighs (Table 3b). The location of the 2^nd^ worst pain problem was more evenly reported at neck/shoulders, arm/hands, and back (Table 3b), while the most common location of the third worst pain problem was back (visit 1) and neck/shoulders (Table 3b).

**Fig. 3:**
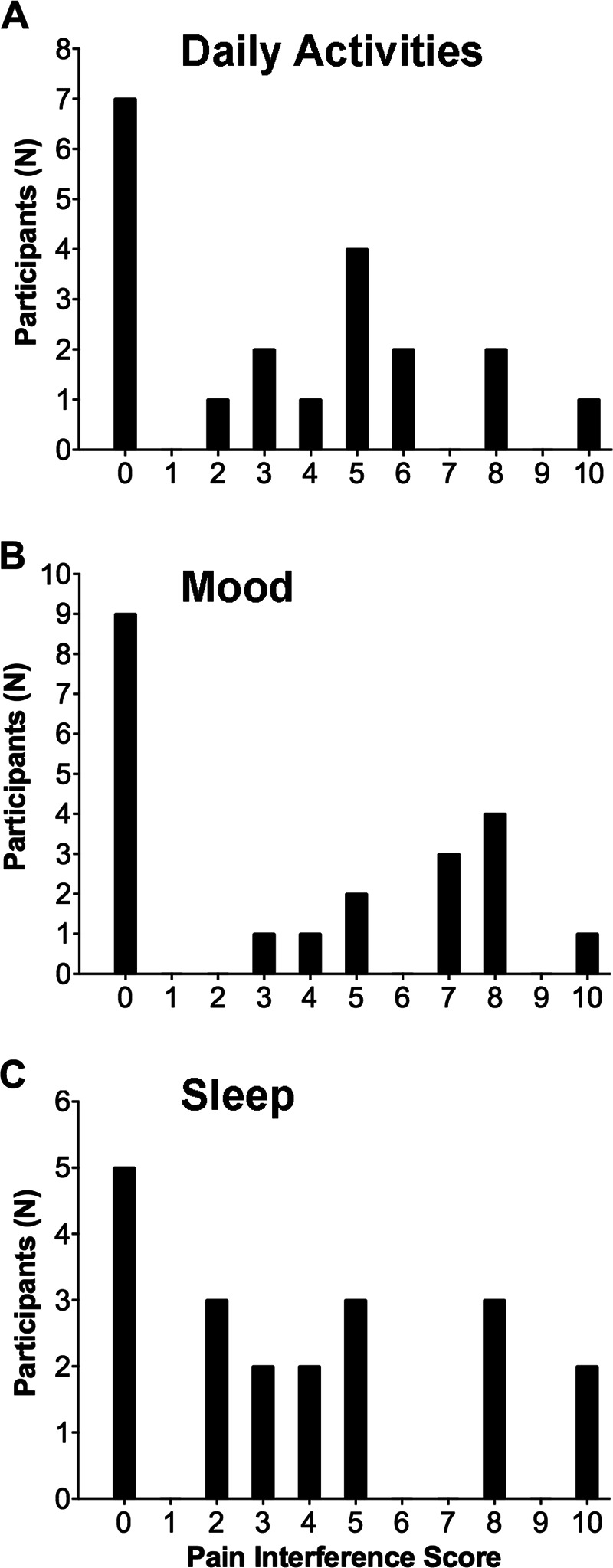
Pain interference: participants were asked to rate their pain interference in response to the questions: **a** In general, how much has pain interfered with your day-to-day activities in the last week? **b** In general, how much has pain interfered with your ability to get a good night’s sleep? **c** In general, how much has pain interfered with your overall mood in the last week? The *x* axis in (**c**) applies to all and shows the pain interference score, (0 is no interference and 10 is the worst interference). The *y* axis shows the corresponding number of participants who responded with that interference score. Data shown are from Visit 1

ISCIPBDS (version 1) included six questions to characterize aspects of pain interference with activities of living, using a 0–10 scale, where 0 is no interference and 10 is extreme interference. Questions in version 1 asked about effects of pain during the past week: (1) how did pain limit activities, (2) how did pain change the ability to participate in recreational and social activities, (3) how much did pain change satisfaction or enjoyment in family activities, (4) how did pain interfere with daily activities in general, (5) how did pain interfere with overall mood in general, and (6) how did pain interfere with the ability to sleep at night? Version 2 retained only the latter three questions on daily activity, mood, and sleep. Answers were recorded for most participants at both study visits, (*N* = 21/31 participants at visit 1 and *N* = ≥18/25 participants at visit 2). In response to the question: “In general, how much has pain interfered with your day-to-day activities in the last week?,” 39% and 22% of participants with recorded responses at visits 1 and 2 said there was no interference (Fig. 3a). In response to the question: “In general, how much has pain interfered with your overall mood in the past week?,” 43% and 22% of participants with recorded responses at visits 1 and 2 said there was no interference (Fig. 3b). In response to the question: “In general, how much has pain interfered with your ability to get a good night’s sleep?”, 24% and 20% of participants with recorded responses at visits 1 and 2 said there was no interference (Fig. 3c).

Although this was a pilot study with a small number of participants, we next examined if the presence, type, intensity, or location of pain correlated with any clinical or demographic variables. Forty percent of participants without pain had complete neurological injury motor status, while 57% among participants with pain. The frequency distribution of mechanism of injury was: Fall (40%), MVA (20%), Sports (40%) for participants without pain, and Fall (29%), MVA (24%), Sports (29%), and others (19%) in the group with pain. The age of participants without pain was 54 ± 16.9 and 55 ± 14.9 (mean ± SEM) in participants with pain. The number of years after injury was 13 ± 12.4 in participants without pain and 17 ± 13.0 (mean ± SEM) in participants with pain. Participants with pain reported an average of 6 ± 2.4 (mean ± SEM) numerical rating scale (NRS) units of pain intensity. Pain presence did not show a significant association with SCI level, neurological injury motor status, or mechanism of injury. There was no difference in the years after injury between participants with or without pain (*p* = 0.503). Average pain intensity was not different between SCI levels (*p* = 0.65), neurological injury motor status (*p* = 0.817), or by mechanisms of injury (*p* = 0.281). There was a negative Spearman correlation coefficient between average pain intensity and years after injury, but this was not statistically significant (*p* = 0.2065). Participants with neuropathic pain showed a negative and significant correlation (*ρ* = −0.84979, *p* = 0.004) between years post-injury and the average pain intensity. Stratifying participants by SCI level, neurological injury motor status, or mechanism of injury also did not show significant correlation among year of post-injury and pain intensity.

### International SCI quality of life data set

Many tools have been used to measure QoL in persons with SCI [17]. The International SCI QoL Basic Data Set asks participants to provide a subjective rating of the past four weeks in three domains: general QoL (overall well-being), satisfaction with physical health, and satisfaction with psychological health. Each domain is ranked on a 0–10 scale, with 0 indicating completely dissatisfied and 10 indicating completely satisfied. Here, we used the International SCI QoL Basic Data Set as compared to a group of similar age able-bodied (AB) persons (*N* = 28). The general QoL score was significantly lower in persons with SCI vs. AB controls (7.2 ± 0.4 vs. 8.5 ± 0.25 mean ± SEM, *p* < 0.007). The satisfaction with physical health score was significantly lower in persons with SCI vs. AB controls (6.3 ± 0.5 vs. 8.1 ± 0.24 mean ± SEM, *p* < 0.005). The satisfaction with psychological health score was significantly lower in persons with SCI vs. AB controls (7.4 ± 0.4 vs. 8.7 ± 0.2 mean ± SEM, *p* < 0.02). This study shows that QoL was significantly lower across all domains of the International SCI QoL Basic Data Set.

Of course, QoL in persons with SCI can be influenced by multiple factors including comorbidities, socioeconomic status, psychosocial support, access to health services, and others. Given that we used both the pain and QoL data sets in the same participants, we next investigated whether QoL scores differed in participants with SCI by pain status. While lower, there was not a significant correlation between pain status and QoL scores: QoL rating of physical health, and satisfaction with psychological health were 8 ± 1.3, 7 ± 2.1, and 8 ± 1.2 in the group without pain and 7 ± 2.6, 6 ± 2.7, and 7 ± 2.6 in the group with pain. The average pain intensity had a negative, but not significant, correlation with general QoL (*ρ* = −0.102. *p* = 0.668, *N* = 20), rating of physical health (*ρ* = −0.392. *p* = 0.088, *N* = 20), and satisfaction with psychological health (*ρ* = −0.177. *p* = 0.454, *N* = 20). Participants with pain showed a significant difference in the rating of physical health by neurological injury motor status (complete vs. incomplete, 7 ± 2.8 vs. 4 ± 1.8, *p* = 0.04), but not in other QoL scales. After stratifying by mechanism of injury, participants in Fall and in MVA groups showed a significant negative correlation between average pain intensity and rating of physical health (Fall: *ρ* = −1.000, *p* < 0.0001, *N* = 5; MVA: *ρ* = −0.889, *p* = 0.044, *N* = 5).

Pain type also did not show any difference in the QoL scales with regard to SCI level, mechanisms of injury, or neurological injury motor status. The Spearman correlations among the years post-injury and the general QoL scale, rating of physical health, and satisfaction with psychological health was not statistically significant, but the results suggested that there might be positive correlations. When participants were stratified by the mechanism of injury, it showed that years post-injury was positively and marginally significantly correlated to the rating of physical health in MVA group (*ρ* = 0.775, *p* = 0.041, *N* = 7). There was no correlation between years post injury and general QoL or satisfaction with psychological health in participants with or without pain, or by pain type.

## Discussion

Clinical and demographic data from participants in this study is generally consistent with data from the NIH SCI Model Systems database: the majority of participants were male, had cervical level injuries and the most common AIS grades were A and D (Table 1). The most common etiologies of injury here were Fall and Sports, the latter of which is not as common nationally as motor vehicle accidents [3]. In this study, the majority of participants reported experiencing pain, including more than one pain problem, consistent with national and international data (Fig. 1) [3, 5, 18–21]. Pain intensity has been reported as moderate to severe across studies and as expected, pain negatively impacts overall QoL in persons with SCI (e.g., [1, 22, 23]). To manage pain symptoms, many people with SCI are prescribed multiple therapies, including gabapentin/pregabalin, and tricyclic antidepressants for neuropathic pain, and physical therapy, exercise, and NSAIDS for nociceptive pain [19, 21]. Concurrent pharmacologic treatments are typical for persons with SCI and in this study more than two thirds of participants received pharmacological treatment for pain, with pregabalin or gabapentin the most common medication taken, along with NSAIDS. This was also recorded in a recent study in Italy using an Italian translation of the ISCIPBDS [24].

Unfortunately, despite taking medications for multiple types of pain, pain symptoms persist after SCI, and here we found that participants were almost evenly divided on whether they reported nociceptive or neuropathic pain as their worst pain problem (Table 3). Nociceptive pain was the most common second and third worst pain problem. A study of pain among individuals with SCI in Switzerland using the International SCI Pain Basic Data Set yielded similar results: 68.9% had some kind of pain in the past week, with the most common pain type nociceptive (musculoskeletal), followed by neuropathic pain (below level), 41.6% [25]. A large study (*N* = 643) of individuals with SCI in Ireland that used the International SCI Pain Basic Data Set similarly found that 76% of respondents used pharmacological treatment for pain, 71% had some kind of pain, with nociceptive and neuropathic pain experienced by 32% and 37% of the respondents, respectively [26].

For the worst pain problem, pain intensity was over 7 on a NRS, which was higher for neuropathic pain at both study visits (Fig. 2). In Ireland, the average pain intensity was 6.3 ± 2.2 [26]. In the Swiss study, average pain intensity over the past week was 6 ± 2 [25]. For the worst pain problem, pain location was most commonly reported here as neck/shoulders, which is consistent with previous literature from the SCI population [27, 28].

As found here, a meta analysis of 42 studies concluded that often there is no correlation between injury completeness or level and pain [1]. A retrospective study from a Swiss pain center that used the ISCIP classification of pain found that 58% of participants (*N* = 66) had nociceptive pain, 53% had at-level and 42% had below-level neuropathic pain [29]. Here, 38% of persons had at-level and 62% of persons had below-level neuropathic pain.

In a study of persons (*N* = 90) followed at 1, 6, and 12 months after SCI, 80% of participants had pain at all time points, 59% had neuropathic and nociceptive pain at 1 year after SCI, and the percentage of individuals with neuropathic pain increased over time [20]. In another study of Siddall and colleagues that continued for five years after SCI, 81% of participants reported pain, of which nociceptive (musculoskeletal) was the most common (59% of participants), and 41% and 34% reporting at-level and below-level neuropathic pain, respectively [18]. Half of participants reported pain of any type within the first three months after SCI and the average time of any type of pain onset was 1.6 years after SCI, with at-level neuropathic pain onset a bit earlier, at 1.2 years after SCI [18]. While we did not address onset of pain here, data from Siddall and colleagues showed that individuals with neuropathic pain within the first six months post SCI were likely to have ongoing severe pain five years after injury [18].

**Fig. 4:**
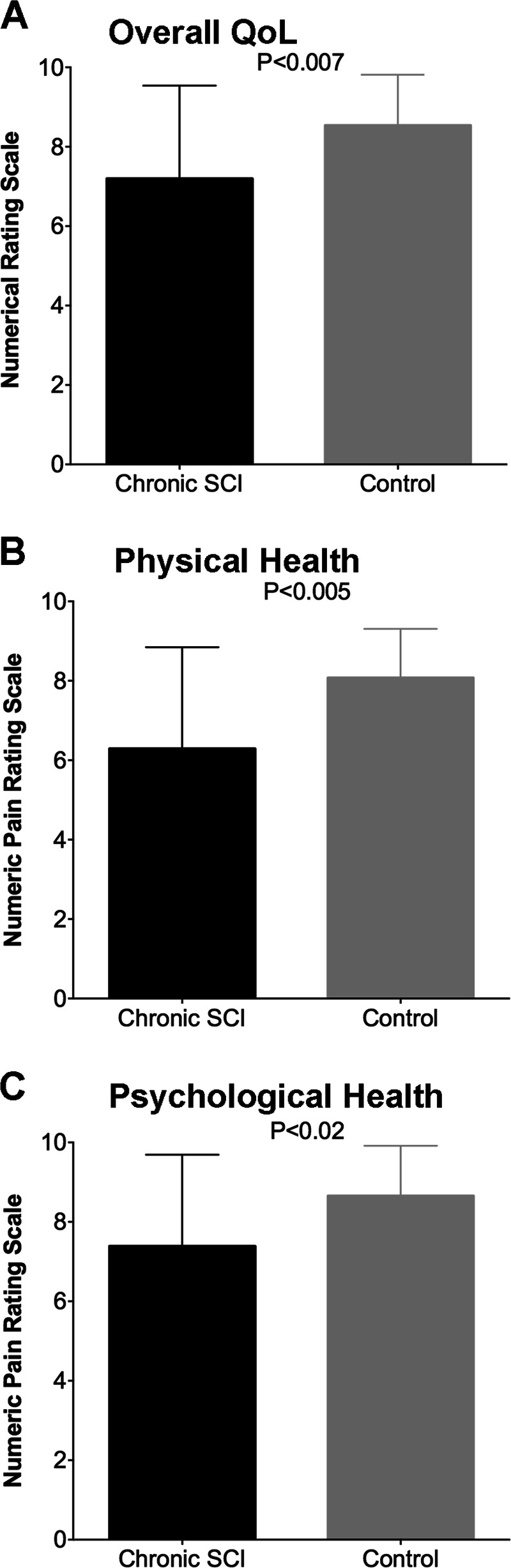
Quality of Life: At visit 1, participants were asked to rate their QoL over the last 4 weeks on a scale of 0–10, where 0 is completely dissatisfied and 10 is completely satisfied. Graphs indicate ratings of: **a** overall QoL, **b** satisfaction with physical health, **c** satisfaction with psychological health, emotions and mood. Data from participants with chronic SCI and AB participants are shown in black and gray, respectively

In addition to pain, SCI often triggers psychosocial changes that can negatively impact QoL [30, 31]. On the ISCIPBDS, pain interference ranged from 0–10 on the different subscales and was highest for most participants on the question related to sleep interference (Fig. 3). Multiple factors, including pain, other medical comorbidities, psychosocial support, community integration, and socioeconomic resources, have been shown to impact QoL after SCI. Using the QoL Basic Data set, QoL scores were lower for participants with SCI than AB persons (Fig. 4), consistent with a recent validity study [14]. A survey study of individuals with SCI showed that the presence and intensity of pain correlated with interference in general activity, mood, mobility, interpersonal relationships, self-care, and other facets of daily living [27]. Other studies demonstrated that pain intensity correlated with pain interference also showed that psychosocial factors such as coping mechanisms, such as catastrophizing contributed to greater pain perception and pain interference [32, 33]. Therefore, as there are many factors that can impact QoL scores, it is not surprising that we did not observe a significant correlation between pain presence or intensity with QoL scores in this relatively small number of participants.

Overall, these data reinforce the notion that despite being treated for pain, most individuals with SCI are living with multiple pain problems and that lower QoL needs to be addressed in individuals with SCI. There are several strengths of this study. To our knowledge, this is the first study to use the International SCI Pain Basic and the QoL Data Sets prospectively in persons with chronic SCI. Data were collected from the same participants at two study visits held six months apart and the clinical/demographic characteristics were generally consistent with national US data. There were several limitations to this study, including the relatively small sample size and incomplete data collection from all participants, including that a minority of participants (19%) did not complete the second study visit.
